# Supplementary material for: AlphaFold predictions of fold-switched conformations are driven by structure memorization
Source: Nat Commun. 2024 Aug 24;15:7296. doi: 10.1038/s41467-024-51801-z (PMC11344769; doi:10.1038/s41467-024-51801-z)
Supplement: Supplementary file 1 — Supplementary Information [file 41467_2024_51801_MOESM1_ESM.pdf]

Supplementary Information for

AlphaFold predictions of fold-switched conformations are driven by structure memorization

Devlina Chakravarty[1], Joseph W. Schafer[1], Ethan A. Chen[1], Joseph F. Thole[1,2], Leslie A. Ronish[1,2], Myeongsang Lee[1], and Lauren L. Porter[1,2,*]

[1]National Center for Biotechnology Information, National Library of Medicine, National Institutes of Health, Bethesda, MD 20894, USA

[2]Biochemistry and Biophysics Center, National Heart, Lung, and Blood Institute, National Institutes of Health, Bethesda, MD, 20892, USA

*Correspondence: porterll@nih.gov

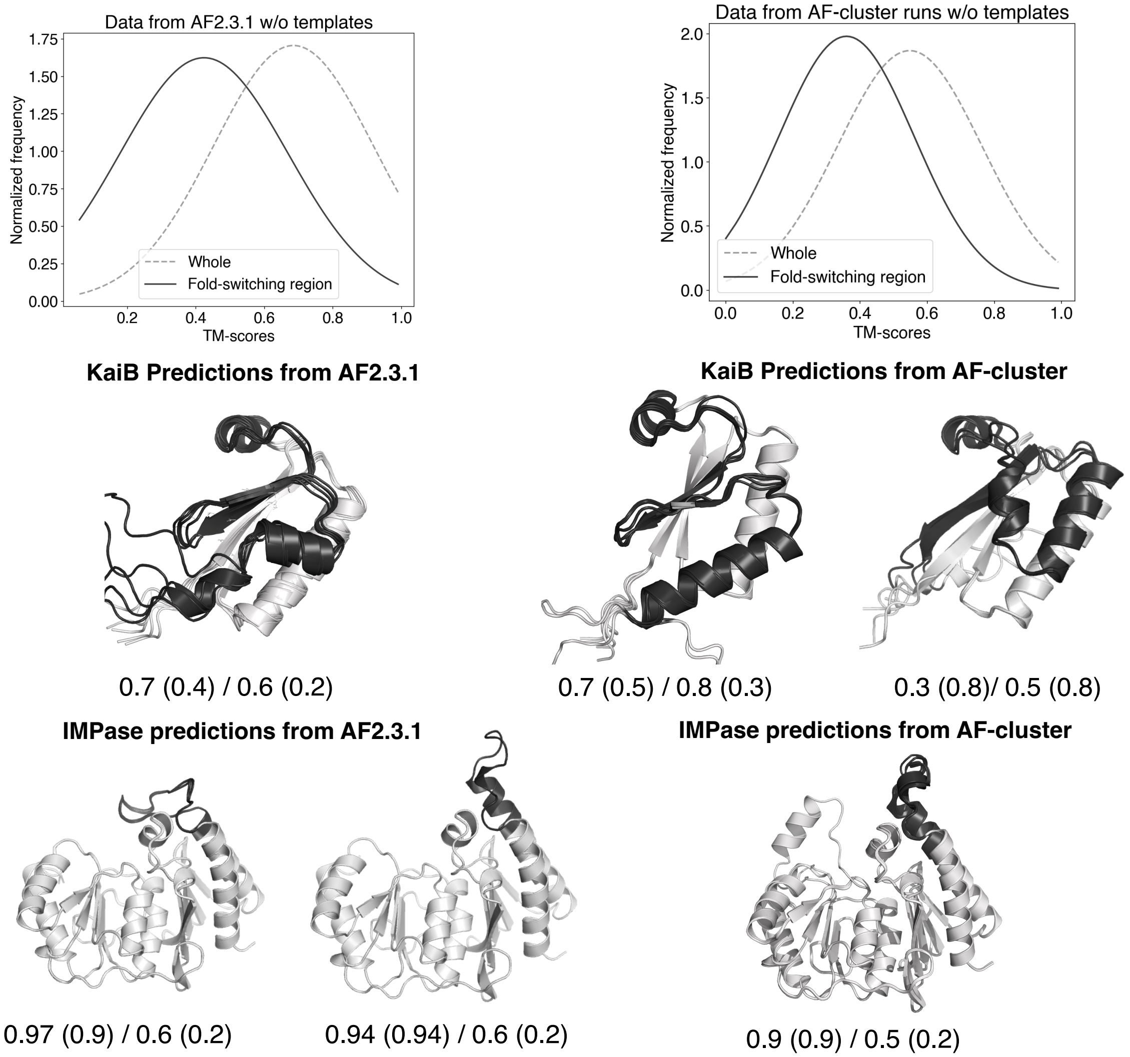

Figure S1. **TM-scores for fold-switching regions represent predictions of fold switchers more accurately than TM-scores of whole proteins**. Distributions of overall vs fold-switching region TM-scores for AF2.3.1 (upper left) and AF-cluster (upper right) demonstrate that whole-protein TM-scores overestimate prediction accuracies corresponding to regions of interest. Examples of predictions from AF2.3.1 and AF-clusters for KaiB and IMPase further demonstrate this point (fold-switching region is highlighted in black and the rest is grey). TM-scores relative to Fold 1 (left of /) and Fold 2 (right of /) are systematically higher for whole proteins (numbers without parentheses) compared to their fold-switching regions (in parentheses). Source data are provided as a Source Data file.

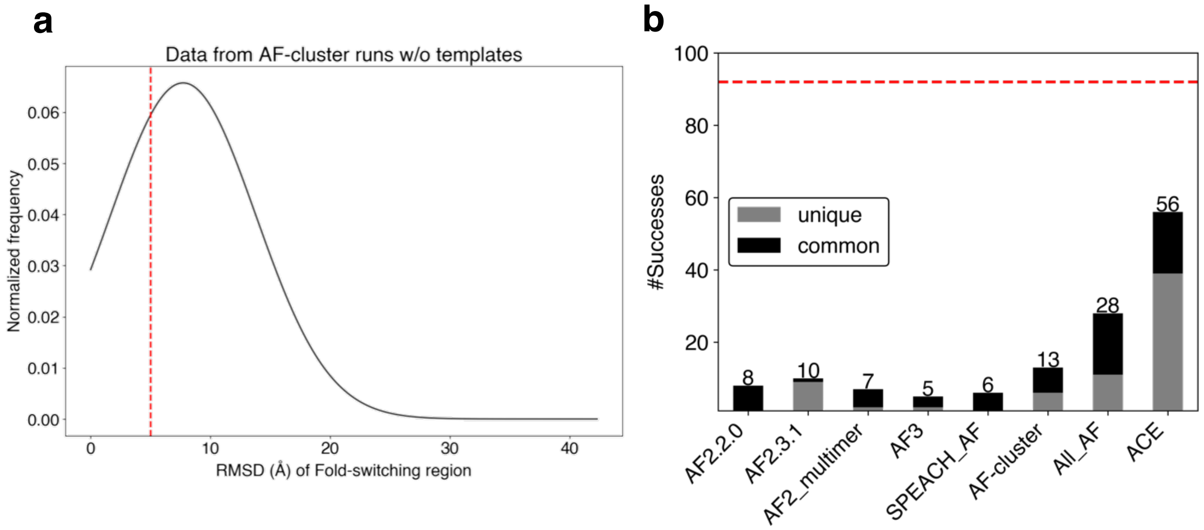

Figure S2. Assessing predictions by RMSD yields results similar to TM-score based assessments. Distribution of RMSD for the fold-switching region of AF-cluster predictions referenced against the fold-switching regions of the most similar experimentally determined structure is presented on the left panel (a), with threshold line at 5 Å. Prediction success measured by RMSD (b, fold-switching RMSD within 5 Å of experiment) yields results similar to TM-score (Figure 1 in main text). Source data are provided as a Source Data file.

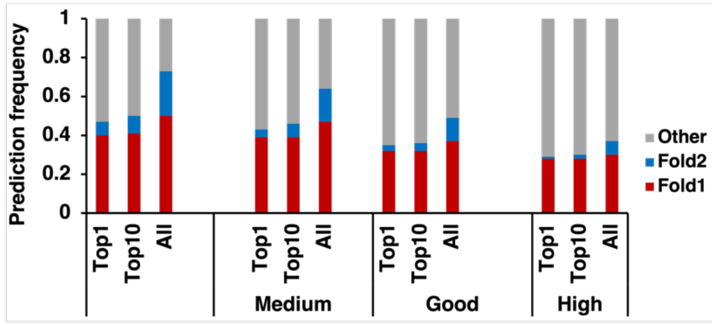

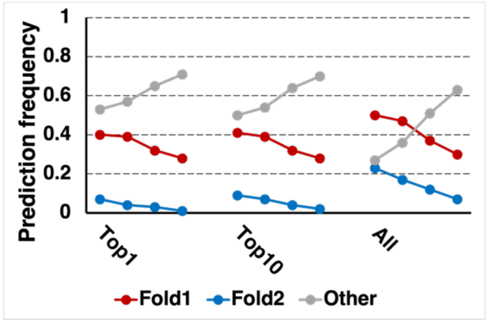

Figure S3. **Ranking by predicted template modeling (pTM) score selects against experimentally observed conformations in favor of experimentally unobserved for AF-cluster predictions.** (a) Bar-plot representation of the Prediction Fraction in Top1,Top10 and All ranked models. (b) Trendline plots showing the change in prediction success in categories –High (pTM>0.9), Good (pTM>0.7), Medium (pTM > 0.6), and All, respectively for Top1, Top10 and All predictions. Neither denotes predictions whose fold-switching regions had TM-scores < 0.6 relative to the experimentally determined structures of both Fold1 and Fold2. Source data are provided as a Source Data file.

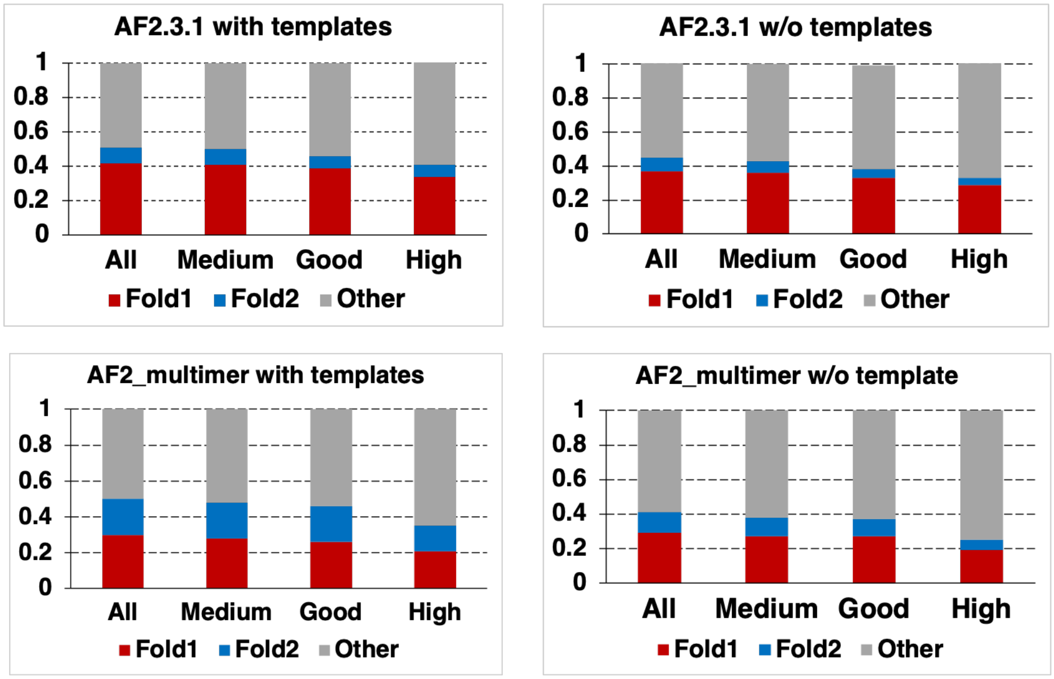

Figure S4. pLDDT scores select against experimentally determined conformations of fold-switching regions in all AF2.3.1. runs. Predictions are ranked by confidence (percentage of residues with pLDDT scores > 70). The categories are defined as - All, Medium (confidence > 70%), Good (confidence > 80%) and High (confidence >90%). Neither denotes predictions whose fold-switching regions had TM-scores < 0.6 relative to the experimentally determined structures of both Fold1 and Fold2. Source data are provided as a Source Data file.

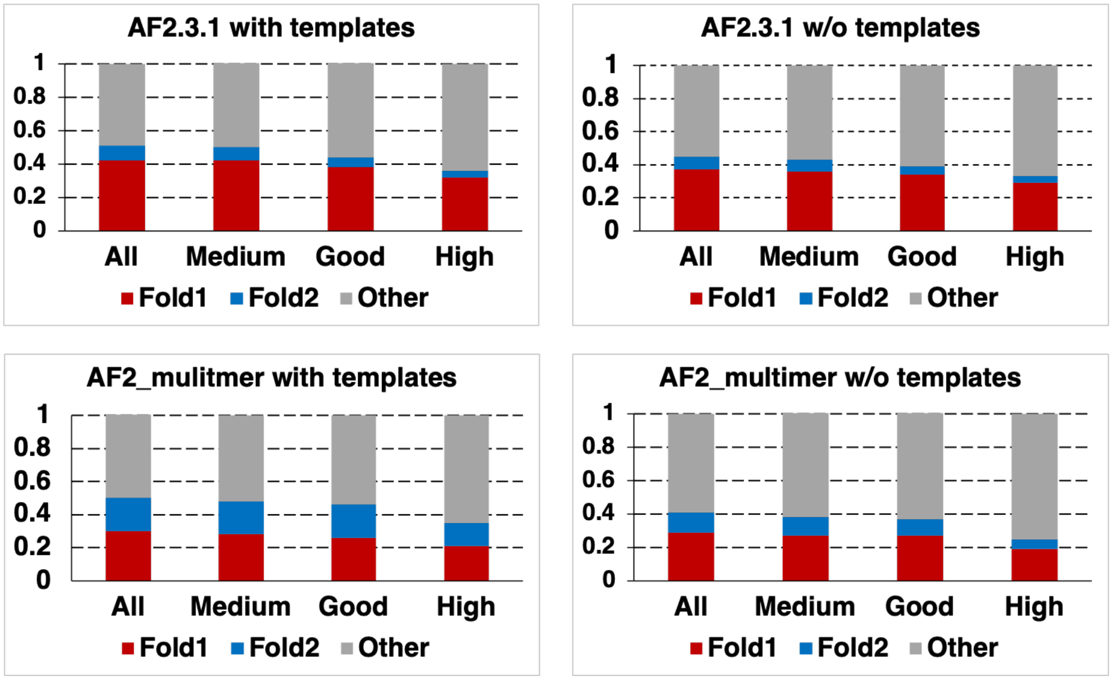

Figure S5. pTM scores select against experimentally determined conformations of fold-switching regions in all AF2.3.1. runs. Predictions are ranked by confidence (pTM score defined as - All, Medium (pTM ≥ 0.6), Good (pTM ≥ 0.7) and High (pTM ≥ 0.8). Neither denotes predictions whose fold-switching regions had TM-scores < 0.6 relative to the experimentally determined structures of both Fold1 and Fold2. Source data are provided as a Source Data file.

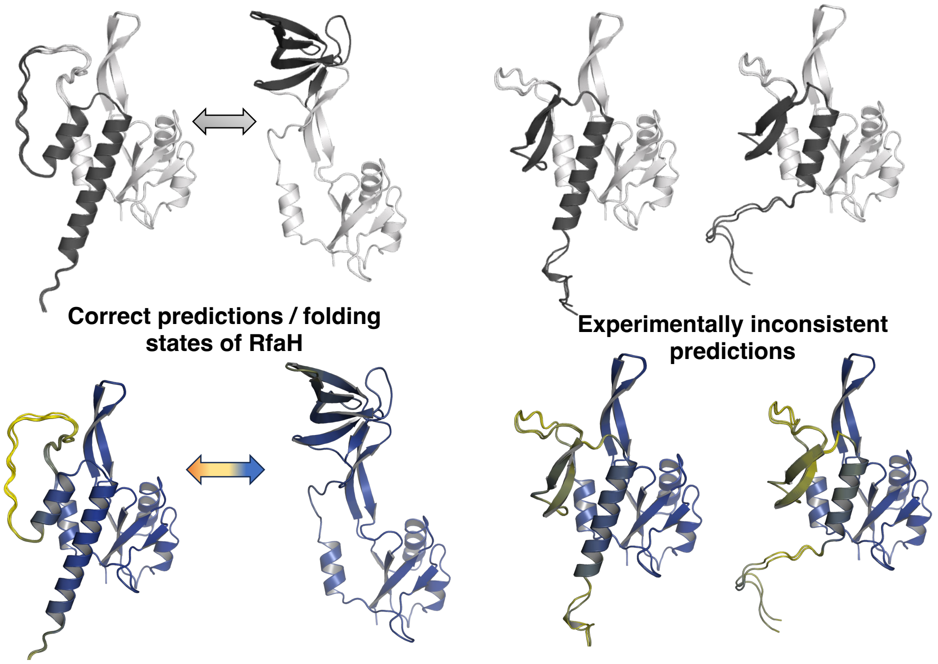

Figure S6. AF2 predicts experimentally inconsistent conformations of RfaH. Models corresponding to experimentally determined structures on left; experimentally inconsistent predictions shown on right. The figures below are colored by pLDDT scores, (color ranging from orange, yellow to blue, corresponding to pLDDT scores from 0 to 100). All predictions were generated from AF2_multimer (run without partner and no templates).

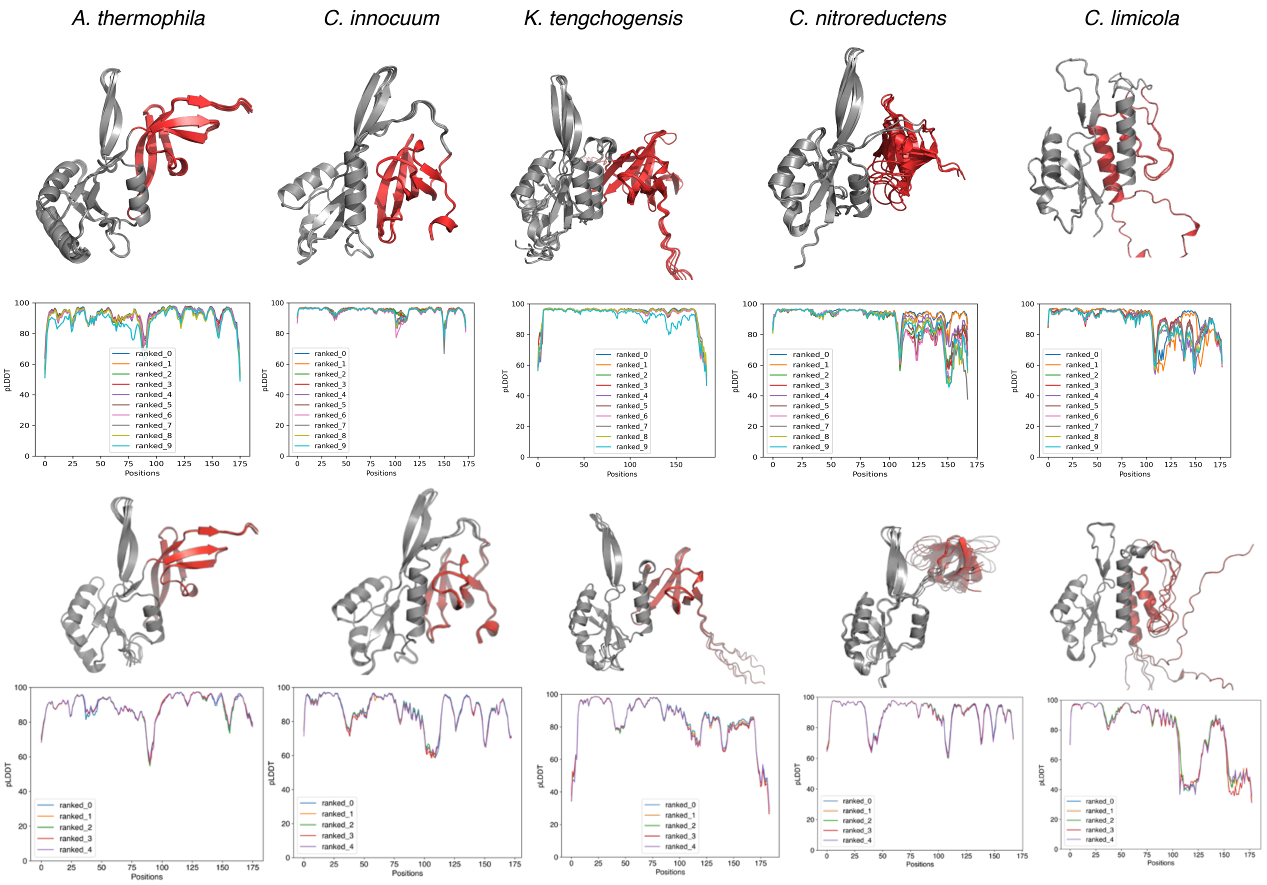

Figure S7. AlphaFold fails to predict the experimentally confirmed helical conformations in the C-terminal domains (CTDs) of 4/5 RfaH homologs. Predictions from AF2.3.1 are in the top panel and the bottom panel has AF3 server predictions. In all variants but *C. limicola*, only β-sheet CTDs (red) are predicted. Structurally conserved N-terminal domains are colored gray. pLDDT scores of all models of each protein generated without templates are shown below their predicted structures. C-terminal domains comprise residues 115-end of protein.

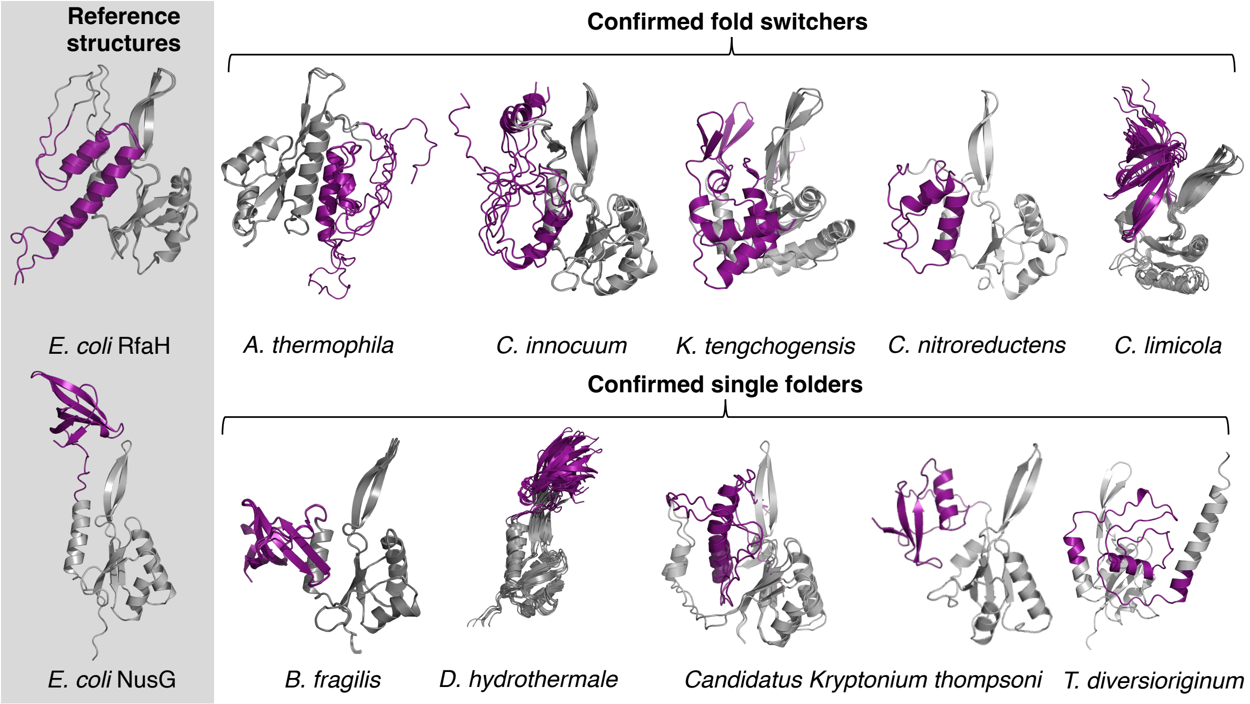

Figure S8. AF-cluster predictions cannot distinguish between RfaH homologs with helical C-terminal domains (CTDs, upper row) and β-sheet C-terminal domains (lower row). Further, pLDDT scores of all helical CTD predictions are low (average ≤50), further indicating that correct and incorrect predictions cannot be distinguished. All CTDs are colored purple; structurally conserved N-terminal domains are gray. Experimentally confirmed reference structures of *E. coli* RfaH and NusG are shown on the left column with the same color scheme.

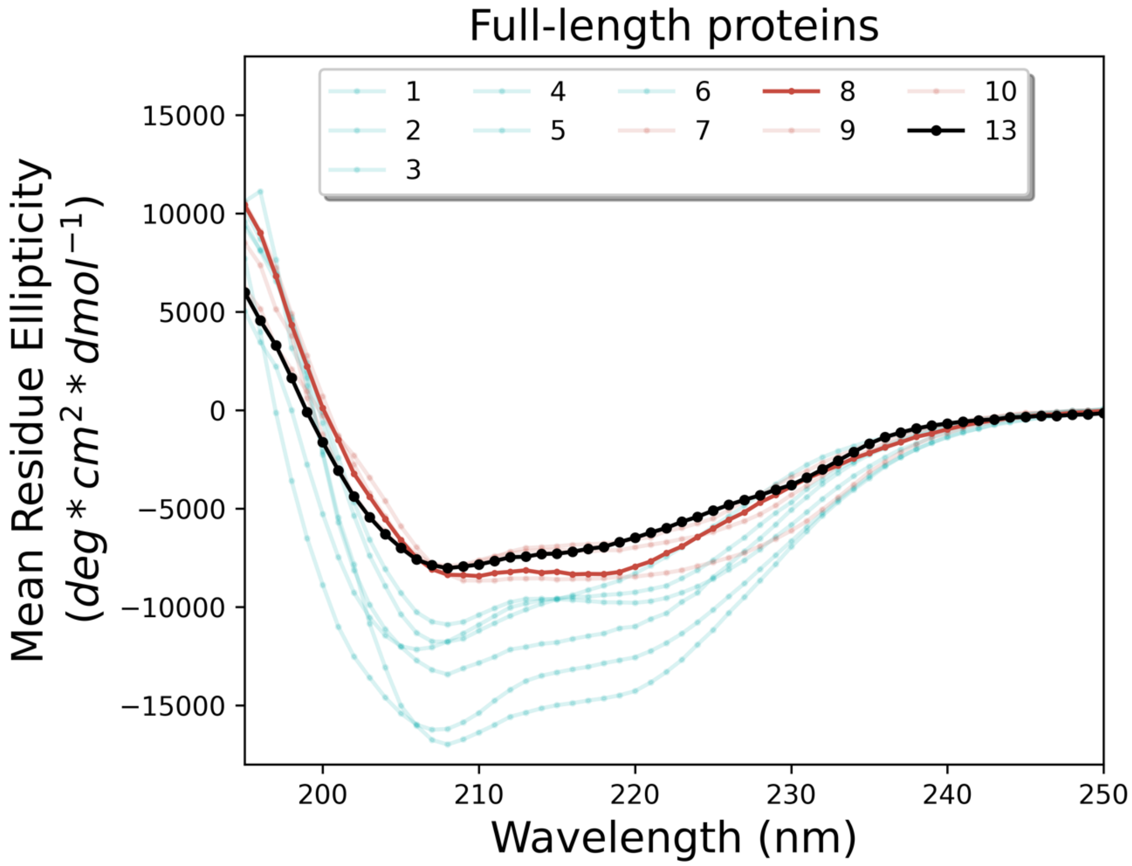

Figure S9. The circular dichroism spectrum of NusG Variant 13 (black) resembles single-folding NusGs with ground state β-sheet folds (red) rather than fold-switching NusGs with ground state α-helical folds (teal). CD spectra of all variants except for 13 were taken from reference 24 in the main text. AF-cluster predicted that it and Variant 8 (bold red) can assume helical folds, inconsistent with experimental evidence. The sequence of Variant 13 (Table S4) inserted into a pET-28a(+) vector was purchased through BioBasic, codon optimized for *E. coli*. Variant 13 was purified using Cytiva Hi-TRAP columns on an ÄKTA Pure at room temperature. Its 6x-His tag was cleaved overnight with biotinylated thrombin (Sigma Millipore) at 4°C while dialyzing in 100 mM potassium phosphate, 10% glycerol (v/v) pH 7.4 using a ThermoFisher dialysis cassette (10 kDa MWCO). The cleaved sample was again run on a Hi-TRAP column, and the unbound flow-through was then concentrated in a Millipore centrifugal concentrator (10 kDa MWCO) and subsequently polished through size exclusion chromatography with a Superdex 70 Increase 10/300 column (Cytiva) and was found to be monomeric. Its CD spectrum was collected on a Chirascan spectrometer (Advanced Photophysics) in 100 mM Phosphate, pH 7.6 at 9µM, 10 scans. Source data are provided as a Source Data file.

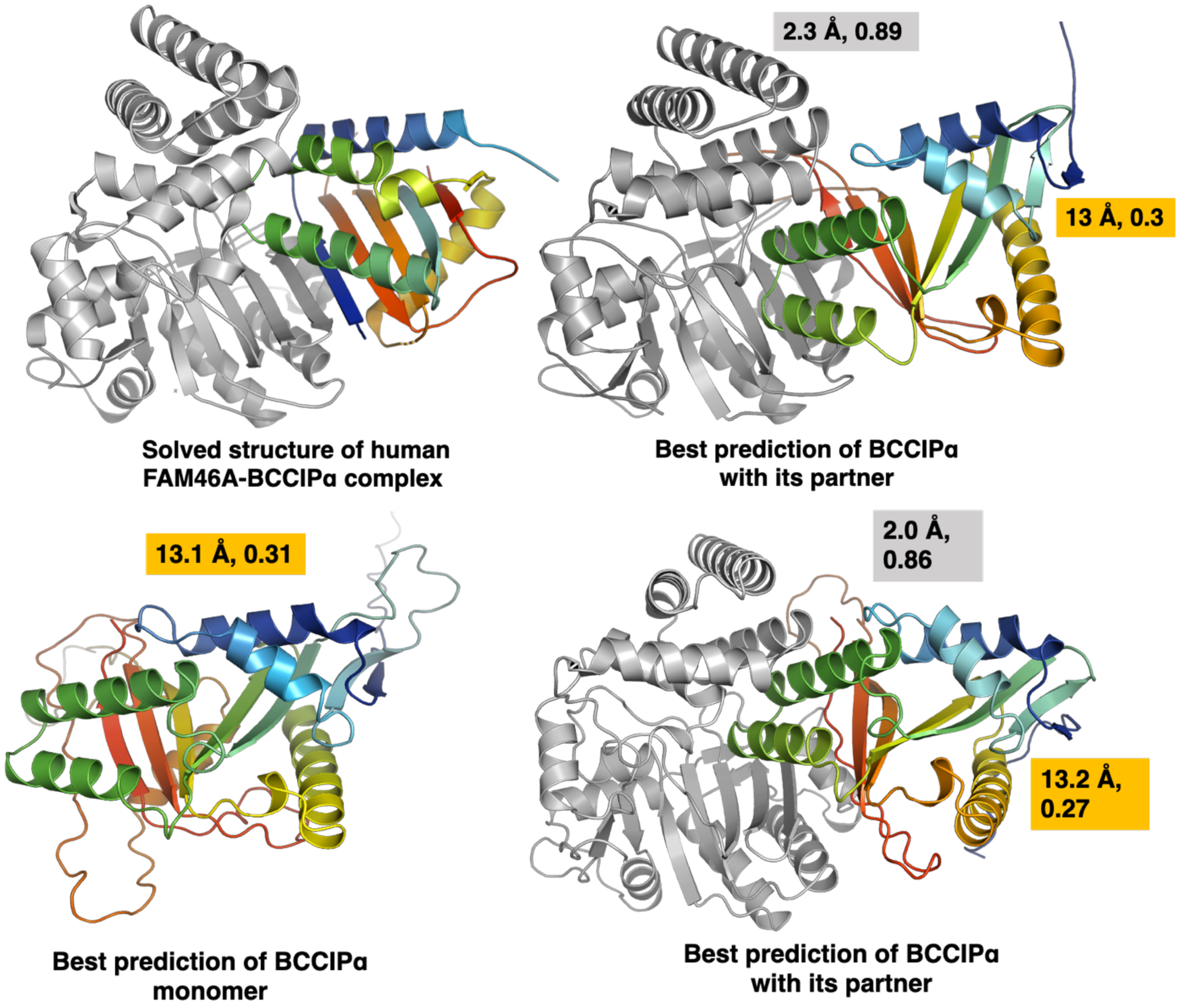

Figure S10. Predicting the structure of BCCIPα with its binding partner using AF2_multimer generated the incorrect BCCIPβ conformation for all models. Structure of the experimentally determined complex (PDB ID: 8EXF, left) differs from all AF2_multimer (upper panel) / AF3 server models (lower panel, ranked 1 shown for all). The structure of BCCIPα's binding partner, FAM46A (gray), was predicted with has high accuracy: TM-score ~0.86 and RMSD ~2.3 Å. Whereas BCCIPα (rainbow N->C, blue->red) was poorly predicted: TM-score ~0.3 and RMSD ~13 Å. The predicted binding interface is also incorrect for the complex predicted.

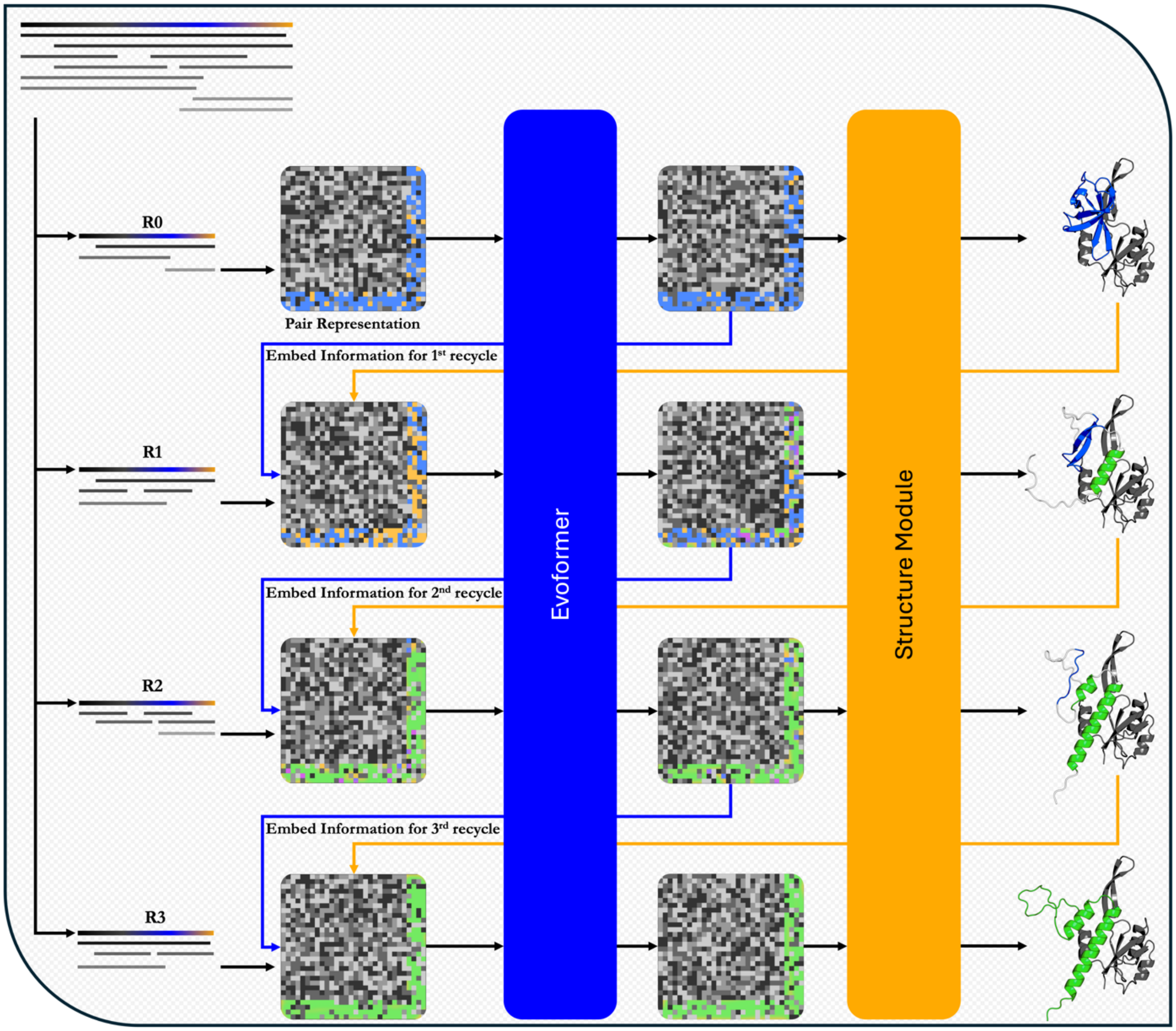

Figure S11. Leveraging AF2s architecture to identify contributions from coevolutionary restraints. A cartoon representation of the initial multiple sequence alignment (MSA) used in AlphaFold2's algorithm is shown in the upper left-hand corner. This MSA is randomly subsampled at each recycling step (R0 through R3). At R0, features of the subsampled MSA are passed into the Evoformer to produce a pair representation, which the Structure Module then maps into a 3D structure (top structure, gray and blue). At subsequent recycling steps (R1-R3), both the pair representation and the 3D structure are inputted into the Evoformer to update the pair representation, which means that the structures predicted from R1-R3 are informed by coevolutionary information, learned protein properties in AF2's weights accessed through recycling, and predictions from the structure module.

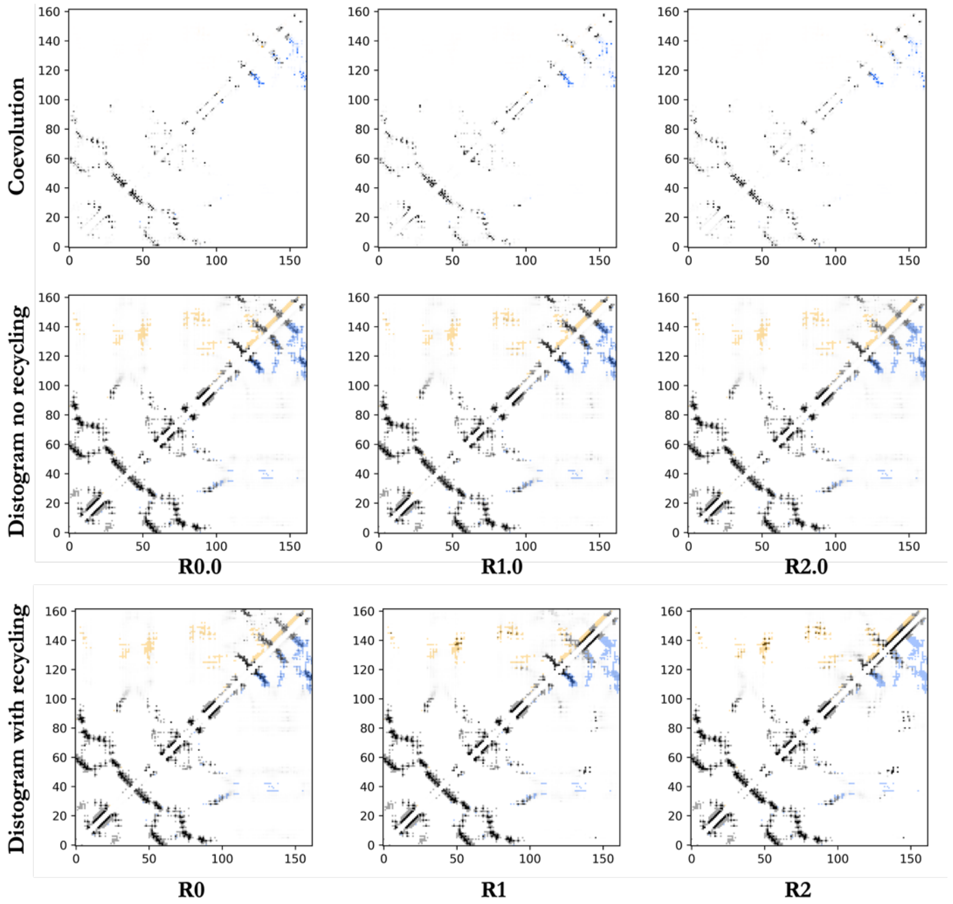

Figure S12. Recycling changes Evoformer's pairwise representation. Although both the coevolutionary signals and the AF2 distograms from each input multiple sequence alignment (MSA) correspond strongly to the β-sheet fold of RfaH's C-terminal domain, subsequent recycling steps shift the prediction from β-sheet to α-helix. Black squares correspond to coevolutionary signals from MSA Transformer (top row), distograms from AlphaFold2 with no recycling (middle row), and distograms from AlphaFold2 with recycling (bottom row). Blue/orange squares are contacts unique to β-sheet/α-helix folds. Thus, overlapping black and blue squares are restraints unique to the β-sheet fold and overlapping black and orange squares are restraints unique to the α-helical fold.

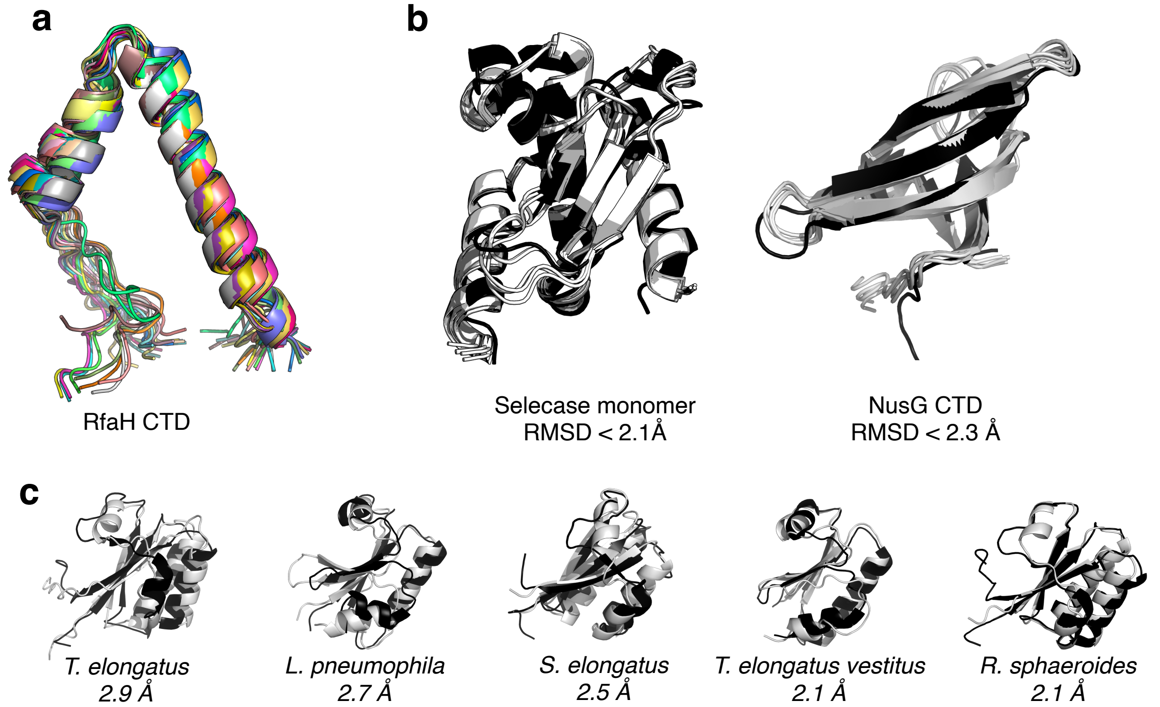

Figure S13. Evidence that AF2 has "memorized" certain conformations during training. This means that it produces structures from single sequences with 0 recycles. (a) AF2 predicts the helical bundle conformation from the sequence of RfaH's isolated C-terminal domain (CTD) from its single sequence in 25/25 instances, though experiments demonstrate that the isolated CTD forms a β-roll fold. (b). The structures of the selecase monomer and NusG CTD produced from single sequences are highly similar to those determined by experiment and likely in AF2's training set. (c). Single-sequence, 0 recycle predictions successfully reproduce all AF-cluster predictions of KaiB variants. 3/5 of these structures were likely in AF2's training set; we hypothesize that AF2 predicts the other two (*T. elongatus vestitus* and *R. sphaeroides*) by associating their sequences with structures memorized during training. For panels (b) and (c), experimentally determined structures are black and predictions are gray. RMSDs were calculated using the cealign function in PyMOL.

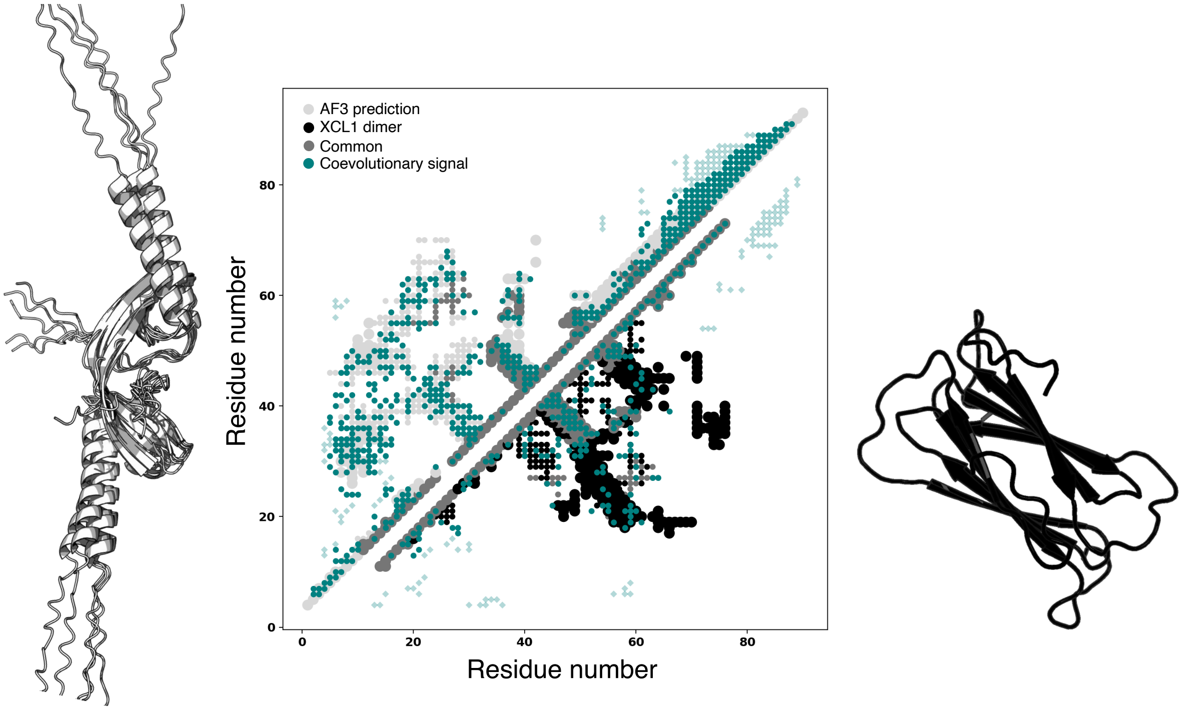

Figure S14. Substantial differences between contacts corresponding to AF3's predicted XCL1 dimer (light gray) and experiment (black). Although coevolutionary signal for both folds can be detected, it seems that AF3 detects monomeric contacts (Figure 5B and gray, upper diagonal) rather than dimeric (black, lower diagonal) and assigns them to interchain rather than intrachain interactions. Contact map (middle) generated from the AF3 prediction and experimentally determined dimeric conformation (PDB ID: 2JP1). Upper diagonal corresponds to contacts unique to the AF3 prediction (light gray, smaller dots correspond to intermolecular contacts; larger to intramolecular), lower diagonal corresponds to contacts unique to the experimentally determined dimeric conformation (black, smaller dots correspond to intermolecular contacts; larger to intramolecular); common contacts medium gray; coevolutionary information inferred from MSA using ACE, (teal).

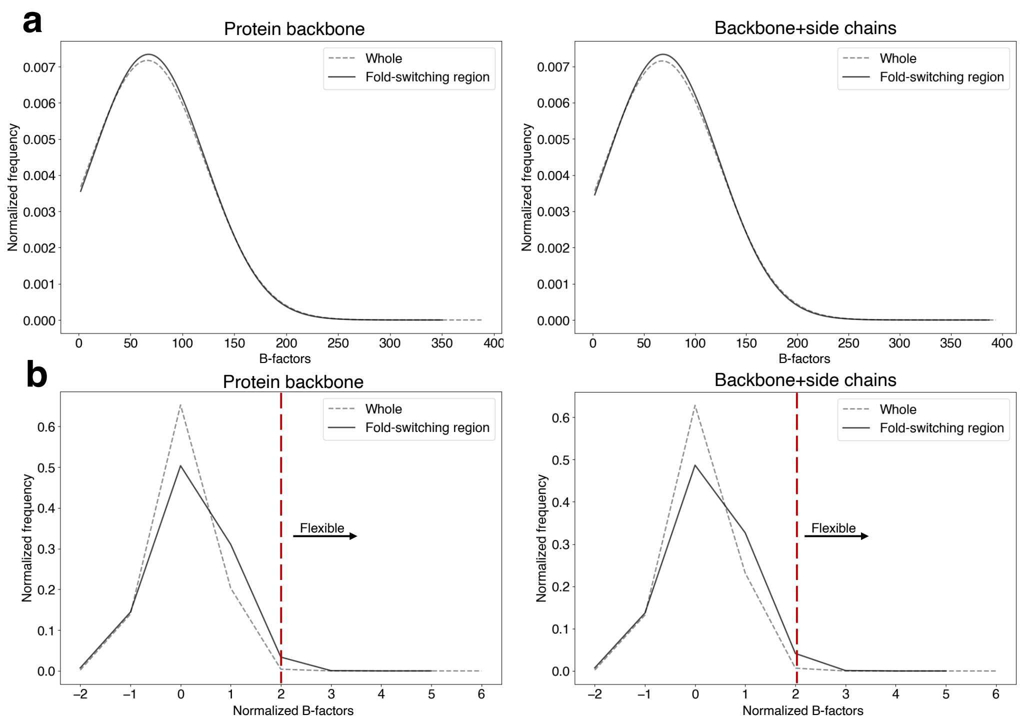

Figure S15. The flexibilities of fold-switching regions are similar to whole solved crystal structures. (a) The raw B-factors of fold-switching protein regions are similar to those of whole fold-switching proteins (fold-switching + single-folding) for both backbone (left, mean ± std Whole: 66.3 ± 55.6, FS: 67.4 ± 54.3) and backbone + side chains (right, mean ± std Whole: 67.8 ± 55.8, FS: 68.8 ± 54.4). (b). The normalized B-factors of fold-switching protein regions do not show significant flexibility relative to the whole protein: only 2% of residues in fold-switching regions have normalized B-factors ≥ 2.0, compared to 1% of residues in the whole protein. Both fold-switching and whole protein B-factors were normalized by B-factors of the whole protein.

Table S1. Sequences of all RfaH homologs tested and depicted in Figures S8 and S9. Fold-switching sequences bold.

Variant	Sequence
C. innocuum	**MMKPWYVLYVMGGKEQKILSLLNKGEDIKAFTPWKEVMHRVQGKRILVKKPLFPSYVFLE<br>TELDPAVFHQKLMLYKSQINGILKELKYEDDISALHTEERAYLEGLMDEEHNVRLSKGEI<br>LDGEVIITEGPLKGYESNIIRIDRHKRRAILNVRMNNQDLQVDVSLEIVKKIESQK**
K. tengchongensis	**MDLNWYVLQTKPKQENLVESYLNLANIEVFNPKIQEIRYIGEKRKKITVLLFPCYVFAKL<br>NPSLFDLVIYTRGVRKILGVNGRPKPIKESIIETIKERIRENSYIYVPENYEEFQLCQGD<br>YVVVVDGPLKGFAGIVERINGSKAIVMLISMDYQVKADIPKFLLRKVDPEILE**
E. coli	**MQSWYLLYCKRGQLQRAQEHLERQAVNCLAPMITLEKIVRGKRTAVSEPLFPNYLFVEFDPE<br>VIHTTTINATRGVSHFVRFGASPAIVPSAVIHQLSVYKPKDIVDPATPYPGDKVIITEGAFE<br>GFQAIFTEPDGEARSMLLLNLINKEIKHSVKNTEFRKL**
C. limicola	**MKVTDRNSCWYAVYVRSRYEKKVHRMFLEKEVEAFLPLLETWRQWSDRKKKVSEPLFRGY<br>VFVNIDMKAEHIKVLDTDGVVKFIGIGKTPSVISSRDIDWIKKLVREPDAVRRIVASLPP<br>GQKVMVTAGPFKGLEGVVVKEGRESRLVVYFDRIMQGIEVSIYPELLSPIHAVGTEEQNE<br>TGFY**
C. nitroreductens	**MESFLNWYLIYTKVKKEDYLEQLLTEAGLEVLNPKIKKTKTVRNKKKEVIDPLFPCYLFV<br>KADLNVHLRIISYTQGIRRLVGGSNPTIVPIEIIDTIKSRMVDGFIDTKSEEFKKGDTIL<br>IKDGPFKDFVGIFQEELDSKGRVSILLKTLALQPRITVDKDMIEKLHN**
A. thermophila	**MSKKWYAIQSKPNKEQALCEQFQSRGIEVFYPQIRVNPVNPRARKIRPYFPGYLFVHVDL<br>DEVGLSVIRWIPFARGVVSFSNEPASVPDNLIEAIRRRVDEVNRAGGELLETLKPGEPVL<br>IQEGPFAGYEAIFDVRLSGKERVRVLIQLLSQRYIPVEMQVGSLKPLKTKNKDKPHPL**
B. fragilis	MSEQQKYWFAARTRDKQEFAIRDSLEKLKTELDLNYYLPTQFVIRQLKYRRKRVEVPVIK<br>NLIFIQATKQDACDISNKYNIQLFYMKDLLTRAMLIVPDKQMQDFIFVMDLDPNGVSFDN<br>DHLSVGSRVQVVKGDFCGVEGELASEANKTYVVIRIAGVLSASVKVPKSYLRVI
C. Kryptonium thompsoni	MARRWYAVRTYSGHENRVKKFIENEIAEGKFKDKIFNVLVPTEKVTVVREGRKKSRVKAF<br>FPGYILIEAEMDDEVKNFIRAVPSVVSFVGPKGNPVPLREDEVERFIGKPEGAELERIDV<br>PFRVGDSVKVIDGPFTDFSGVVQEVNSEKMKLKVMINIFGRKTPVELDFTQVEIEK
E. coli	MSEAPKKRWYVVQAFSGFEGRVATSLREHIKLHNMEDLFGEVMVPTEEVVEIRGGQRRKS<br>ERKFFPGYVLVQMVMNDASWHLVRSVPRVMGFIGGTSDRPAPISDKEVDAIMNRLQQVGD<br>KPRPKTLFEPGEMVRVNDGPFADFNGVVEEVDYEKSRLKVSVSIFGRATPVELDFSQVEK<br>A
D. hydrothermale	MRMDEGLSRSGGDRVAKQWYIVHTYSGFEHRVKAALQERIKAAGKEEYFGQILVPTEKVV<br>ELVKGERKSSSRKFYPGYIVVEMELNDETWHLVRHTPKVTGFIGSQERPIPLSEEEANAI<br>IQQMEEGIQKPRPKYQFEKGEEVRVVDGPFASFNGVVEQVIPEKGKVRVLVTIFGRSTPV<br>ELDFVQIQRL
T. diversioriginium	MYLQKPVYKWYAIYTKANNEKKVFDRLKEENIECYLPLKKTLRQWSDRKKWVDLPLFRCYVF<br>VKVSYIEYFRALRIPGVVYYVSFGGEPQSIPNNQIEYIKAIVQQTEKEIEVNYKNIRKGSEC<br>EVLVGPLKGIKGEVVRISGQSRLLIRLASMGVSLNVNISKDEIKLIKNKATRTAQKKYSSLD<br>RIPYKKSGASVY
