# Supplementary material for: AlphaFold predictions of fold-switched conformations are driven by structure memorization
Source: Nat Commun. 2024 Aug 24;15:7296. doi: 10.1038/s41467-024-51801-z (PMC11344769; doi:10.1038/s41467-024-51801-z)
Supplement: Supplementary file 3 — Description of Additional Supplementary Files [file 41467_2024_51801_MOESM3_ESM.pdf]

Description of Additional Supplementary Files

File Name: Supplementary Data 1

Description: The dataset of 92 fold-switchers on which AF2 was benchmarked. PDB IDs corresponding to both conformations, as well as the amino acid sequence of the fold-switching regions of each protein, are listed.

File Name: Supplementary Data 2

Description: Successful predictions of fold-switchers using each AF2 implementation. Reports from each implementation are shown on individual pages of the Excel spreadsheet.

File Name: Supplementary Data 3

Description: List of biomolecules included in all AF3 runs.

File Name: Supplementary Data 4

Description: Fold-switchers identified with different plDDT cut-offs.

File Name: Supplementary Data 5

Description: Fold-switchers identified with different pTM cut-offs.

File Name: Supplementary Data 6

Description: Ranking of targets by Easy and Complex

File Name: Supplementary Data 7

Description: Number of models generated by AF-cluster for each RfaH variant.
